# Long-Term Psychosocial Impacts of the COVID-19 Pandemic on Older Adults

**DOI:** 10.1093/geroni/igaf122.2308

**Published:** 2025-12-31

**Authors:** Riva Rainier, Jaclyn Bergstrom, Rebecca Daly, Colin Depp, Danielle Glorioso, Anthony Molina

**Affiliations:** University of California San Diego, San Diego, California, United States; University of California San Diego, San Diego, California, United States; University of California San Diego, San Diego, California, United States; University of California San Diego, San Diego, California, United States; University of California San Diego, San Diego, California, United States; University of California San Diego, La Jolla, California, United States

## Abstract

The psychosocial impact of the COVID-19 pandemic on older adults is not fully understood. This study aims to explore the differences in emotional measures pre- and post-pandemic to assess the emotional burden of the COVID-19 pandemic on this population. We hypothesize that older adults will have a significant decrease in emotional wellbeing status post pandemic, with the highest impact on the 75+ older adult population. UCSD Successful Aging Evaluation (SAGE) longitudinal study is a prospective cohort study with a focus on the cognitive and emotional aspects of aging. This analysis utilized validated emotional measures: anxiety (BSIA), depression (CESD), self-compassion (NSCS), resilience (CDRS), and loneliness (UCLA Loneliness Scale). 248 community-dwelling participants were found eligible to be included in this study. Across all participants we observed a significant increase in anxiety (p = 0.0403), depression (p = 0.0707), and loneliness (p = 0.0111) and a significant decrease in self-compassion (p = 0.0441) and resilience (p = < 0.0001). The 50-74 population had higher levels of anxiety (p = 0.0055), depression (p = 0.0189), and loneliness (p = 0.0317) pre-pandemic than the 75+ population. Post-pandemic, the 75+ population experienced a greater change in emotional characteristics with significant changes in anxiety (p = < 0.0001), depression (p = < 0.0001), loneliness (p = 0.0099), self-compassion (p = 0.0006), and resilience (p = < 0.0001) with no significant changes in the 50-74 population. The 75+ population was significantly more affected by the psychosocial impact of the pandemic, indicating greater vulnerability of this population. Uncovering the emotional vulnerability of this population is critical to promote greater awareness and provide insight into potential intervention strategies to aid in full emotional recovery.

